# *In Vitro* Selection of a Single-Stranded DNA Molecular Recognition Element against *Clostridium difficile* Toxin B and Sensitive Detection in Human Fecal Matter

**DOI:** 10.1155/2015/808495

**Published:** 2015-02-05

**Authors:** Ka Lok Hong, Eamonn Maher, Ryan M. Williams, Letha J. Sooter

**Affiliations:** ^1^Department of Basic Pharmaceutical Sciences, West Virginia University, 1 Medical Center Drive, P.O. Box 9530, Morgantown, WV 20506, USA; ^2^Molecular Pharmacology & Chemistry Program, Memorial Sloan Kettering Cancer Center, 1275 York Avenue, New York, NY 10065, USA

## Abstract

Toxin B is one of the major virulence factors of *Clostridium difficile*, a bacterium that is responsible for a significant number of diarrhea cases in acute care settings. Due to the prevalence of *C. difficile* induced diarrhea, rapid and correct diagnosis is crucial in the disease management. In this study, we have employed a stringent *in vitro* selection method to identify single-stranded DNA molecular recognition elements (MRE) specific for toxin B. At the end of the 12-round selection, one MRE with high affinity (*K*_*d*_ = 47.3 nM) for toxin B was identified. The selected MRE demonstrated low cross binding activities on negative targets: bovine serum albumin, *Staphylococcus aureus* alpha toxin, *Pseudomonas aeruginosa* exotoxin A, and cholera toxin of *Vibrio cholera*. A modified sandwich ELISA assay was developed utilizing the selected ssDNA MRE as the antigen capturing element and achieved a sensitive detection of 50 nM of toxin B in human fecal preparations.

## 1. Introduction

Toxin B is a virulence factor secreted by* Clostridium difficile*, an obligate anaerobic, spore-forming, Gram-positive bacillus bacterium [1].* Clostridium difficile* induced diarrhea accounts for more than 300,000 or almost 30% of all cases of diarrhea in acute care settings [2, 3]. It also causes prolonged hospital stays and therefore increased cost burden in the health care system [4]. It has been reported that the cost of* C. difficile* infections is between $436 million and $3.2 billion per year in the USA [4, 5]. In addition to economic burdens, the mortality rates of* C. difficile* infections have also increased from 5.7 per million in 1999 to 23.7 per million in 2004 [6].

*C. difficile* produces two major exotoxins: toxin A and toxin B. Toxin B has been shown to be 1000 times more toxic than toxin A. While both toxins are considered to be the cause of* C. difficile* colitis, all toxin-producing strains of* C. difficile* produce toxin B [7]. Upon colonization of toxic strains of* C. difficile* in the colon, the produced toxins deactivate GTPases, such as Rho and Rac, disrupt cytoskeleton and signal transductions, and result in cell rounding and loss of cell structures, leading to host inflammatory responses [8].

Due to the problems associated with* C. difficile* infection (CDI), early and accurate diagnosis is important for disease management and patient survival [9]. Currently, diagnostic tests for CDI are stool culturing, cell cytotoxicity neutralization assay, enzyme immunoassay (EIA) for toxins A and B, detection of* C. difficile* glutamate dehydrogenase (GDH), and polymerase chain reaction (PCR) detection of* C. difficile *genes [10]. Culturing diagnosis is very sensitive, but the turnaround time can be up to 3–5 days [11]. GDH testing is very sensitive but is not specific and requires additional EIA for toxin A and/or toxin B [11, 12]. There are multiple commercial EIA kits for toxin A/B detection on the market, but their sensitivities vary and they may not be available in all countries [13]. PCR test has a rapid turnaround with good sensitivity and specificity but does not detect the presence of virulence factors and the cost associated with the test may limit its usage [11, 14]. A confirmed diagnosis of CDI usually requires positive results from two or three of the available tests [15, 16]. When the results of these tests are combined, they are sensitive and specific, but excessive cost and turnaround time are major drawbacks. Therefore, it is important to identify new diagnostic techniques that can address some of these limitations.

One potential method of addressing problems associated with toxin B testing is through molecular recognition and detection. Molecular recognition elements (MREs) are defined to have high specificity and affinity toward user defined targets. Molecules such as single-stranded oligonucleotides, small peptides, antibody fragments, and full length antibodies can all participate in molecular recognition and have been studied in different types of biosensors [17–20]. MREs are identified by an* in vitro* selection process called systematic evolution of ligands by exponential enrichment (SELEX), which was first described by the Gold laboratory in 1990 [21]. Nucleic acid MREs are usually selected from a large random library consisting of 10^13^ to 10^15^ different single-stranded DNA (ssDNA) or RNA molecules. The library is enriched through repeated cycles of incubation with the desired target and subsequent removal of molecules that bind to undesired targets. At the end of the selection process, the diversity of the MRE library is decreased to the point that one or a few candidate MREs can be identified for affinity and specificity screening against the target of interest.

In this study, we applied a stringent SELEX scheme to obtain a ssDNA MRE that binds to toxin B with high affinity and specificity [22–24]. The selection scheme was designed to eliminate MREs that bind to negative targets that are likely to coexist in the target environment. Bovine serum albumin (BSA) was chosen to be the first negative target based on its similarity to human serum albumin and its prevalence as a blocking agent in assay applications [25]. Alpha toxin of* Staphylococcus aureus* and exotoxin A of* Pseudomonas aeruginosa* are virulence factors of common nosocomial infections, which have the likelihood to coinfect hospitalized patients [26–28]. Cholera toxin of* Vibrio cholerae* is the causative agent of cholera induced watery diarrhea, which symptomatically mimics CDI [29]. In addition to the selection and characterization of the toxin B-specific ssDNA MRE, a modified enzyme-linked immunoassay (ELISA) has been developed which utilizes the identified ssDNA MRE. The assay was able to show the detection of toxin B in human fecal samples at nanomolar concentrations. This work shows the potential of using ssDNA MREs in diagnostic applications [30–32].

## 2. Materials and Methods

### 2.1. *In Vitro* Selection of Toxin B-Specific MREs

**Figure 1:**
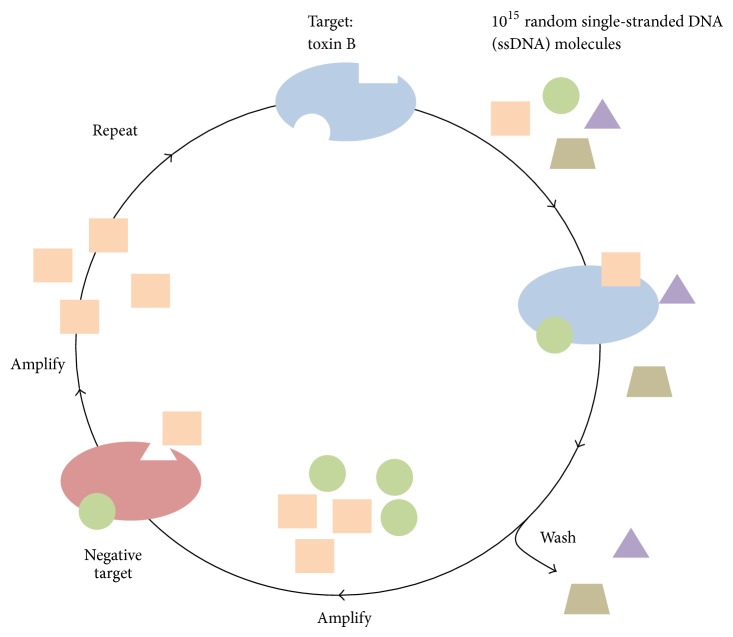
Illustration of the SELEX process. The SELEX process begins with 10^15^ ssDNA molecules and incubation with the target toxin B. Those that bind to toxin B are amplified and subsequently incubated with negative targets. Those that do not bind to negative targets are retained and amplified, thus completing one round of* in vitro* selection.

**Figure 2:**
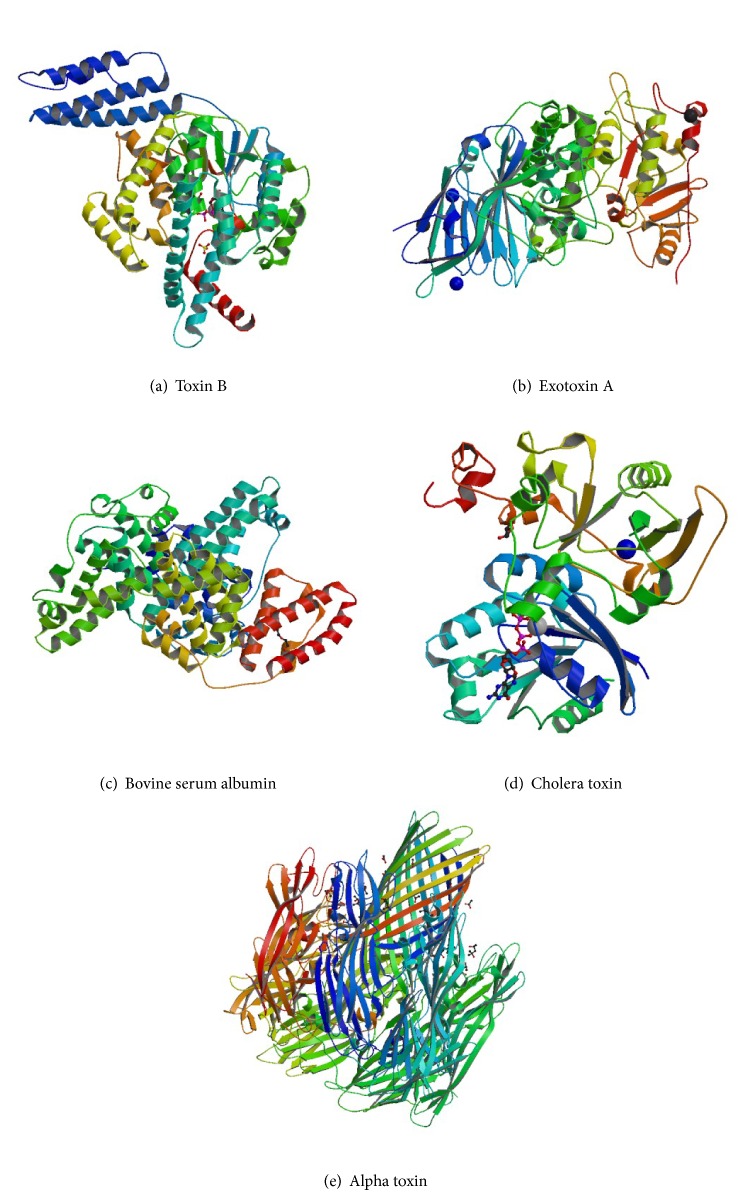
Structures of targets used in the SELEX scheme and cross binding assays. (a) Ribbon structure of the selection target,* Clostridium difficile* toxin B (PDB 2BVM, 270 kDa) [54]. ((b), (c), (d), and (e)) Ribbon structure of* Pseudomonas aeruginosa* exotoxin A (PDB 1IKQ, 66 kDa) [55], bovine serum albumin (PDB 4F5S, 66.5 kDa) [56],* Vibrio cholerae* cholera toxin (PDB 2A5D, 84 kDa) [57], and* Staphylococcus aureus* alpha toxin (PDB 3ANZ, 33 kDa) [58], used in negative rounds of selection and cross binding assays.

The selection process began with a single-stranded DNA (ssDNA) library consisting of 10^15^ different molecules designed by our laboratory as previously described (Figure 1) [22]. In brief, the library, termed RMW.N34, consists of two 23-base constant regions for polymerase chain reaction (PCR) amplification flanking a 34-base random region (commercially synthesized by Eurofins MWG Operon; Huntsville, AL, USA). A total of 12 rounds of SELEX were performed (Table 1) to identify ssDNA molecules that bound specifically to toxin B and not to negative targets (Figure 2).

Lyophilized toxin B (List Biological Laboratories; Campbell, CA, USA) was reconstituted in pure water and covalently immobilized to carboxylic acid-coated magnetic beads (Dynabeads M-270 carboxylic acid) (Life Technologies; Grand Island, NY, USA) via an amidation reaction using N-hydroxysulfonyl succinimide (sulfo-NHS) (Pierce; Rockford, IL, USA) and 1-ethyl-3-(3-dimethylaminopropyl) (EDC) (Pierce; Rockford, IL, USA) according to manufacturer's protocol.

For positive rounds, 6 *μ*L of immobilized target was incubated with ssDNA library in 200 *μ*L of selection buffer composed of 100 mM sodium chloride, 20 mM Tris-HCl, and 2 mM magnesium chloride (1x selection buffer, SB) at room temperature with rotation (8 RPM). After incubation, the immobilized target and solution were separated using a magnet. Unbound ssDNA in solution was removed. Immobilized target/DNA complexes were washed three times with 200 *μ*L of SB and resuspended in 100 *μ*L of SB. This bound DNA served as a template for PCR amplification. The PCR conditions were as follows: bound ssDNA, 400 nM forward and biotinylated reverse RMW.N34 primers (Eurofins MWG Operon; Huntsville, AL) (forward: 5′-TGTACCGTCTGAGCGATTCGTAC-3′, biotinylated reverse: 5′-biotin-GCACTCCTTAACACTGACTGGCT-3′), 250 *μ*M deoxynucleotide triphosphates, 1x GoTaq reaction buffer (Promega; Madison, WI, USA), 3.5 units* Taq *polymerase, and pure water. Thermal cycling conditions were as follows: denature at 95°C for 5 minutes; cycle at 95°C for 1 minute, 63°C for 45 seconds, and 72°C for 1 minute; and final extension temperature at 72°C for 7 minutes [22]. A large-scale 3 mL amplification was carried out after each round of positive and negative selection. This selection procedure for the immobilized toxin B target was performed for rounds 1–6, each with decreasing incubation time.

After PCR amplification, amplified dsDNA was purified with the IBI PCR purification kit (IBI Scientific; Peosta, IA, USA) according to the manufacturer's protocol. Eluted dsDNA containing the biotinylated reverse strand was subjected to single-strand separation and ethanol precipitation of the forward strand as previously described [22]. This procedure was performed after each positive and negative round of selection.

For negative rounds, multiple negative targets were covalently immobilized to carboxylic acid-coated magnetic beads as described above. Immobilized negative targets were incubated with the enriched ssDNA library in the same conditions as positive rounds. However, after magnetic separation, unbounded ssDNA in solution was used as template for PCR amplification. This selection procedure for immobilized negative targets was performed for rounds 2–6.

Competitive elution with free toxin B in solution was performed beginning in round 7 positive. The enriched ssDNA library was first incubated with immobilized toxin B as described above. After magnetic separation and washes, toxin B at a concentration of 20 *μ*g/mL in 100 *μ*L of 1x SB was added to magnetic beads and incubated for 5 minutes and then subjected to magnetic separation. The solution containing ssDNA bound to free toxin B served as PCR template. This procedure was performed for rounds 7–12 positive, each with decreasing time of incubation and target concentrations.

Similarly, competitive elution with negative targets in solution was performed beginning in round 7 negative as outlined above. However, ssDNA molecules bound to the immobilized target were resuspended in 100 *μ*L of 1x selection buffer and served as PCR template. This procedure was performed for rounds 7–11 negative.

### 2.2. Cloning and Sequencing of Toxin B-Specific MREs

In order to analyze the ssDNA library for consensus binding sequences, the library was cloned and sequenced following rounds 3 negative, 6 negative, 9 negative, and 12 positive. Identical procedures were performed as previously described [22]. In brief, the library was amplified with nonbiotinylated primers, and the fresh PCR product was ligated into the pCRII vector (Invitrogen; Carlsbad, CA, USA) and cloned into competent* E. coli* according to the manufacturer's protocol. The cloned plasmid was extracted and purified with AxyPrep Plasmid Miniprep kit (Axygen; Union City, CA, USA) and subsequently sequenced with the M13R primer by commercial source (Eurofins MWG Operon). A total of 30–80 randomly selected clones were sequenced and analyzed.

### 2.3. Toxin-Specific MRE Sequence Alignment and Analysis

Sequences were first grouped into superfamilies by analyzing common tetranucleotide sequences from the variable regions of sequenced clones. The largest superfamily was then chosen for alignment and divided into smaller subfamilies. All sequences in the subfamilies were analyzed for their predicted secondary structures, the predicted Gibbs free energy values of those structures, and the percent homology. These parameters were considered in the choice of candidate sequences.

### 2.4. Toxin B MRE Binding Assays with Surface Plasmon Resonance

One candidate sequence from the round 12 library was chosen for further characterization. The candidate sequence was designated as R12.69. The secondary structure was predicted by the Mfold DNA web server using the following conditions: 25°C, 100 mM Na^+^, and 2 mM Mg^2+^ [33]. Commercially synthesized 5′-amino-C6 modified R12.69 (5′-amino-C6 indicates a primary amino group attached to the 5′ end of the oligonucleotide with a six-carbon spacer between the two) (Eurofins MWG Operon; Huntsville, AL, USA) was used for surface plasmon resonance (SPR) affinity assays. Both in-house produced and commercially purchased CM5 (GE Healthcare; Piscataway, NJ, USA) SPR sensor chips were used for binding assays.

Home-made gold chips were fabricated from glass slides (12 mm × 10 mm) coated with a 2 nm titanium adhesion layer and a 45 nm gold layer. Metals were deposited using a Temescal BJD-2000 system (Edwards Vacuum; Phoenix, AZ) with an Inficon XTC/2 deposition controller (East Syracuse, NY). The home-made gold chips were first cleaned in 100% ethanol under sonication for 5 minutes and then immersed in a solution of 10 mM 11-mercaptoundecanoic acid (11-MUA) (Sigma; St. Louis, MO) and 10 mM triethylene glycol mono-11-mercaptoundecylether (PEG3) (Sigma) in a 1 to 5 ratio overnight under argon for the formation of the self-assembled monolayer. Subsequently, gold chips were rinsed in 100% ethanol and pure water, blown dry with nitrogen, and assembled onto carrying cartridges for SPR binding assays using a Biacore X100 (GE Healthcare; Piscataway, NJ, USA).

Both in-house produced and CM5 purchased SPR sensor chips were activated by injecting 100 mM N-hydroxysulfonyl succinimide (sulfo-NHS) (Pierce; Rockford, IL, USA) and 400 mM 1-ethyl-3-(3-dimethylaminopropyl) (EDC) (Pierce; Rockford, IL, USA) at a 1 to 1 ratio to both active (flow cell 2) and reference (flow cell 1) flow cells at a flow rate of 5 *μ*L/min for ten minutes. An immobilization buffer composed of 100 mM sodium chloride, 20 mM potassium phosphate, and 2 mM magnesium chloride, pH 7.4, was used as the running buffer. Then, 300 *μ*L of 100 nM 5′-amino-C6 modified R12.69 in immobilization buffer was injected into the active flow cell at a flow rate of 5 *μ*L/min, followed by 10-minute injection of 1 M ethanolamine-HCl pH 8.5 into both active and reference flow cells in order to inactivate unreacted sensor surface. Maximum levels of immobilization were obtained for affinity analyses.

Single cycle kinetics assays were performed to determine the affinity of R12.69 to toxin B. The 1x selection buffer was used as running buffer during kinetics assays. Toxin B at various concentrations (20 nM, 40 nM, 60 nM, 100 nM, and 200 nM) in 1x SB was injected into both flow cells at a flow rate of 30 *μ*L/min for 120 seconds with a dissociation time of 150 seconds. Control and baseline adjusted sensorgram responses were analyzed with the Biacore X100 evaluation software (GE Healthcare; Piscataway, NJ, USA). A 1 : 1 kinetics model was used to determine the equilibrium dissociation constant (*K*_*d*_). This binding assay was performed in triplicate.

### 2.5. Toxin B Fluorescence Cross Binding Assays

To determine the cross binding activities of the selected MRE, 5′FAM modified R12.69 was purchased from Eurofins MWG Operon. The assay was performed as previously described with slight modifications [34]. Toxin B, exotoxin A (List Biological Laboratories; Campbell, CA, USA), alpha toxin (List Biological Laboratories; Campbell, CA, USA), cholera toxin (List Biological Laboratories; Campbell, CA, USA), and BSA at 40 nM in 90 *μ*L of 50 mM carbonate/bicarbonate buffer (pH 9.6) were added into individual wells of a 96-well Nunc C8 LockWell MaxiSorp microplate (Pierce; Rockford, IL, USA). Wells containing 1x SB with 0.05% Tween-20 served as the negative background control. The plate was placed on a shaker and incubated at 4°C overnight for protein coating (500 RPM). Subsequently, wells were blocked with 90 *μ*L of 1x SB with 0.05% Tween-20 for 1 hour and washed with the same blocking buffer 3 times. Fluorescently labeled R12.69 was diluted to 100 nM in 90 *μ*L of 1x SB. It was then added to each well and incubated at room temperature for 1 hour. Unbound R12.69 was then aspirated and followed by washing with 1x SB 5 times. Finally, 90 *μ*L of 1x SB was added to each well and the fluorescence emission was measured by a Synergy 2 microplate reader with excitation at 490 nm and emission at 520 nm (Biotek US; Winooski, VT). Fluorescence measurements were normalized to 90 *μ*L of 100 nM fluorescent MRE in 1x SB as described previously [22]. Protein target sets were performed in triplicate and control well sets in duplicate. All data was averaged and standard deviations were calculated. A one-tailed *t*-test was performed to determine the statistical significance in difference of the means (*P* ≤ 0.05).

### 2.6. Toxin B-Specific MRE Modified ELISA Assays

Commercially synthesized 5′-amino-C6 modified R12.69 was used as the toxin B capturing element in a modified sandwich ELISA assay. First, 40 nM of 5′-amino-C6 modified R12.69 in immobilization buffer (100 mM sodium chloride, 20 mM potassium phosphate, and 2 mM magnesium chloride, pH 7.56) was denatured at 95°C for 5 min and cooled to room temperature. Then, 100 *μ*L of the ssDNA was added to individual wells of a maleic anhydride activated plate (Pierce; Rockford, IL, USA) and incubated overnight with shaking at room temperature (500 RPM). Each well was then blocked with 0.1% BSA in 1x SB for 1 hour and washed three times with wash buffer containing 0.1% BSA, 0.05% Tween-20 in 1x SB at room temperature to remove nonimmobilized ssDNA.

Normal human fecal samples (Lee Biosolutions; St. Louis, MO, USA) were reconstituted in 1x SB at 1 g to 20 mL ratio and then centrifuged at 5000 ×g for 10 minutes to collect fecal solution. Toxin B was spiked into 100 *μ*L of prepared fecal solution and 100 *μ*L of 1x SB, respectively, at a final concentration of 50 nM and served as active testing samples. Blank wells without immobilized ssDNA served as the first negative control, and 100 *μ*L of 1x phosphate buffer solution, 100 *μ*L of 1x SB, and 100 *μ*L of fecal solution in wells with immobilized ssDNA served as the second negative control. All samples were added to individual wells and incubated for 1 hour with shaking at room temperature (500 RPM).

**Figure 3:**
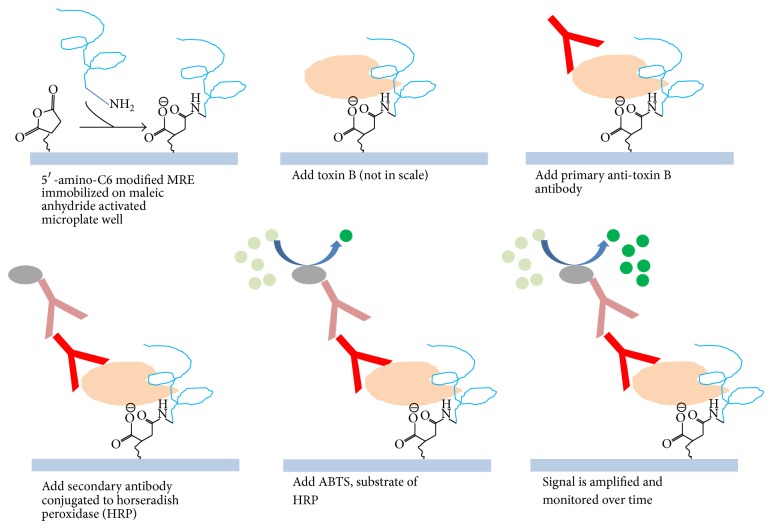
Illustration of the modified ELISA assay. The ssDNA MRE was used as the capturing element in the modified sandwich ELISA assay and signal is amplified by using secondary antibody conjugated to horse radish peroxidase (HRP).

After sample incubation, wells were washed three times with wash buffer to remove unbound toxin B. Then, 100 *μ*L of chicken anti-toxin B primary antibody (List Biological Laboratories; Campbell, CA, USA) at a 1 to 400 dilution ratio in wash buffer was added to each well and incubated for 30 minutes at room temperature with shaking (500 PRM). The primary antibody was then aspirated and each well was washed three times as outlined above. A secondary goat anti-chicken antibody conjugated to horseradish peroxidase (Pierce; Rockford, IL, USA) at a 1 to 500 dilution ratio was added and incubated for 30 minutes at room temperature with shaking (500 RPM). Lastly, all contents were aspirated and washed five times with wash buffer to remove nonspecifically bound antibodies (Figure 3). Additional negative controls were wells without antibodies and wells with only primary antibodies. Assays were performed in duplicate.

ABTS substrate (Pierce; Rockford, IL, USA) was added to individual wells according to the manufacturer's protocol. Absorbance at 410 nm and 650 nm was measured in a Synergy 2 microplate reader using Gen5 1.06 software (Biotek US; Winooski, VT, USA) in two-minute increments. All the data was averaged and standard deviations were calculated. A two-tailed Student's *t*-test was used to determine statistical differences at *P* < 0.05.

## 3. Results and Discussions

### 3.1. Identification of a Toxin B-Specific MRE

Twelve rounds of SELEX were carried out to identify ssDNA MREs specific to toxin B (Table 1). This SELEX scheme is designed to enrich ssDNA MREs that bind to toxin B in solution and exclude ssDNA molecules that bind to BSA, alpha toxin, exotoxin A, and cholera toxin, which are likely to coexist in the target environment. Multiple negative selection rounds were performed to enhance the specificity of the ssDNA library. After every three complete rounds of selection (rounds 3, 6, 9, and 12), 30–80 random sequences were selected and analyzed.

**Figure 4:**
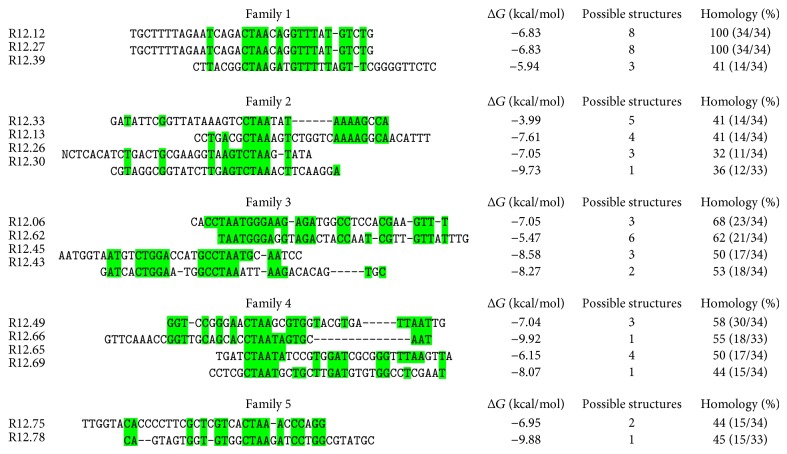
Sequence families of the round 12 library. Only the variable region is shown in the aligned subfamily of the CTAA superfamily. MRE sequences are aligned to the CTAA tetranucleotide sequence. Highlighted regions represent sequence homology shared in the subfamilies. Δ*G* represents the Gibbs free energy values. Possible structures indicate the number of predicted structures from the Mfold web server [33]. Percent homology is calculated from highlighted nucleotides divided by the length of the sequenced variable region.

**Figure 5:**
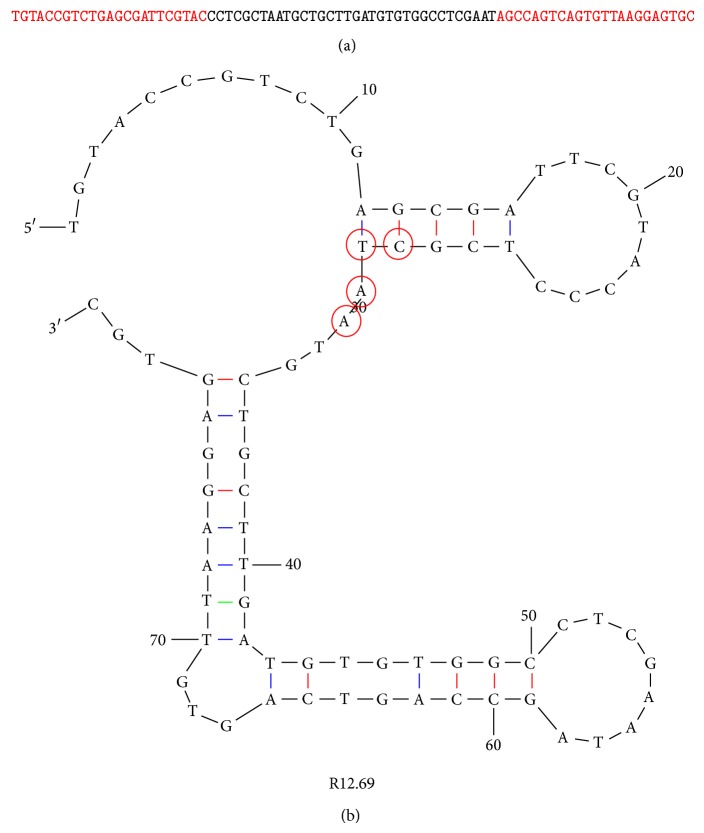
Secondary structure and sequence of R12.69 ssDNA MRE. (a) ssDNA sequence of toxin B MRE R12.69. The red portions indicate the constant regions for primer attachment, and the black portion indicates the variable region. (b) Mfold prediction of R12.69 secondary structure. Highlighted sequence, CTAA, represents most common tetranucleotide sequence in the variable regions of the 43 sequences obtained from the round 12 library, and it is used as the center for sequence alignment in Figure 4 [33].

In the round 12 ssDNA library, 43 sequences were successfully obtained and analyzed. The largest superfamily contained 17 sequences. All 17 sequences were further aligned based on the common tetranucleotide sequence (CTAA) and divided into five smaller subfamilies (Figure 4). It is to be noted that one sequence R12.62 only contained TAA trinucleotide; however it shared large homology within the subfamily, and therefore it was also included in the analysis. The CTAA tetranucleotide was not found in the constant regions, and therefore the constant regions did not participate in the family analysis and were omitted in the figure presentation. However, previous studies showed that the constant regions of the MRE sequence can be involved in their functional secondary structures; thus they were not ignored in the overall decision of choosing candidate sequences [35–39]. Two sequences, R12.12 and R12.27, were identical. However, the sequences were not chosen for further characterization based on the relatively higher Gibbs free energy value (Δ*G*) (indicating lower stability) and multiple possible secondary structures. Sequences R12.30, R12.66, and R12.78 had the lowest Δ*G* values, but their variable regions did not sufficiently participate in the formation of stem-loop structures according to the Mfold predictions. Only one sequence, R12.69, had one possible predicted structure, with a relatively low Δ*G* value (−8.07 kcal/mol), and sufficient stem-loop structures formed from the variable region (Figure 5). Therefore, R12.69 was chosen for further characterization.

### 3.2. Affinity and Specificity of Toxin B-Specific MRE

**Figure 6:**
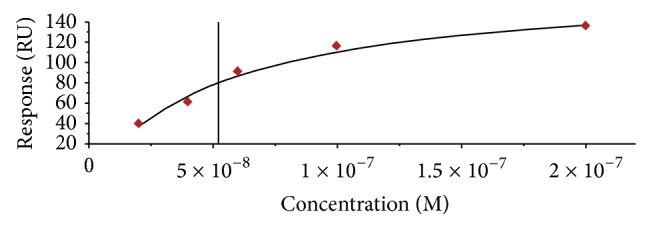
Affinity measurements of R12.69. A representative SPR affinity saturation curve of R12.69 with 1 : 1 binding fit. The averaged equilibrium dissociation constant and standard error of three SPR measurements is 47.3 ± 13.7 nM.

Surface plasmon resonance was used to determine the affinity of R12.69. Single cycle kinetics analysis was performed on both home-made and commercial CM5 SPR sensor chips. This type of assay was chosen instead of multicycle kinetics because there was no need to predetermine the regeneration condition of the sensor chip. This type of assay has also been used in previous studies to determine the binding affinities of nucleic acid MREs [40, 41]. Two assays were performed on CM5 sensor chip and one assay was performed on home-made sensor chip. There were negligible differences between the equilibrium dissociation constants (*K*_*d*_) obtained from both types of sensor chips. The average *K*_*d*_ value from the three assays was determined to be 47.3 ± 13.7 nM (Figure 6). It is to be noted that the MRE was immobilized covalently onto the sensor chip surfaces, which was different than most of the previous studies [40, 42–44]. The surfaces of both home-made and CM5 sensor chips were negatively charged under a neutral to basic running buffer (IM buffer), and the electrical repulsion between the negatively charged DNAs may lead to variable levels of ligand immobilization and different levels of maximum SPR response unit. However, analyte and ligand binding was saturated in all three independent assays (Figure 5), thus validating the use of covalent linkage for MRE immobilization in SPR analysis. The determined dissociation constant (*K*_*d*_) was comparable to other MREs targeting bacterial toxins [34, 45, 46].

The cross binding activity of R12.69 was determined by fluorescence plate assay. The ssDNA MRE preferably binds to toxin B greater than other negative targets in the selection scheme (Table 2). The binding of R12.69 to toxin B is 2.2 times higher than chorea toxin (*P* = 0.0497), 5.4 times higher than alpha toxin (*P* = 0.0117), 4.7 times higher than exotoxin A (*P* = 0.0130), and 5.8 times higher than bovine serum albumin (*P* = 0.0106). It is to be noted that both cholera toxin (84 kDa) and alpha toxin (33 kDa) were introduced only once in the negative selection scheme (Table 1). However, the selectivity over alpha toxin is more than double that of cholera toxin. It is likely that the ssDNA library was enriched to bind preferably to large globular protein targets (M.W. of toxin B = 270 kDa) during early selection rounds. Other negative targets were introduced multiple times in the negative selection scheme and therefore their respective cross binding activities were sufficiently decreased. This result validates that multiple negative targets and the competitive elution strategy employed in our stringent SELEX method can greatly enhance the specificity of ssDNA MREs.

### 3.3. Diagnostic Application of Toxin B-Specific MRE

**Figure 7:**
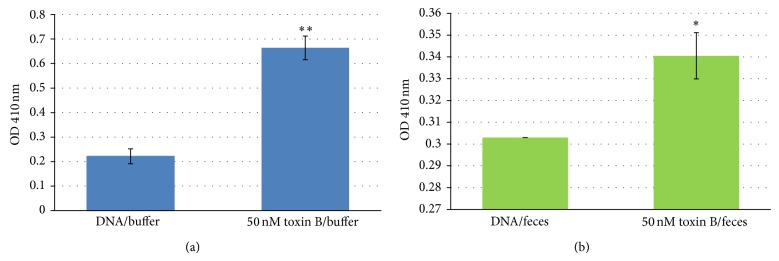
Modified ELISA assays of toxin B. Data from one modified sandwich ELISA assay with absorbance measured at OD 410 nm. Absorbance levels presented are subtracted from background levels of blank wells without immobilized DNA (negative control). Error bars represent 2x standard deviations of 2 sample replicates. (a) Statistical significance levels with respect to DNA in buffer background (without toxin B) of *P* < 0.01 are designated by ∗∗. (b) Statistical significance levels with respect to fecal background (without toxin B) of *P* < 0.05 are designated by ∗. Buffer: 1x selection buffer; feces: 1 g/20 mL 1x selection buffer.

A modified sandwich ELISA assay was developed in this study to investigate the translational potential of R12.69. Reproducible detection of 50 nM toxin B spiked in human fecal solutions was achieved compared to control in 8 minutes after HRP substrate incubation (*P* < 0.05) (Figure 7). It is to be noted that fecal matter is a complex matrix, which contains multiple macromolecules and ions [47]. Meanwhile, the three-dimensional structure of nucleic MREs is highly dependent on the temperature, pH, and ionic strength of the binding condition and these structures are related to their binding abilities [48]. This modified ELISA assay demonstrated the robustness of R12.69 in complex biological matrices. Previous studies have identified ssDNA MREs specific for bacterial toxins and similar ELISA assays were developed for toxin detection [34, 49]. However, both ELISA detections were not tested in clinically relevant samples, which is necessary for translation. Recently, slow off-rate modified binding elements (SOMAmer by SomaLogic, Inc.; Boulder, CO, USA) specific for toxins B and A and binary toxin of* C. difficile* have been identified with subnanomolar affinities [50]. The authors also reported sensitive detection of toxin B at picomolar concentrations in multiple assays, though fecal preparations were not tested in all of the assays. A previous study reported that fecal toxin B levels in patients with CDI ranged from approximately 26 ng/mL to 25 *μ*g/mL [51]. The current MRE modified ELISA assay can detect toxin B level at 50 nM (1.35 *μ*g/mL), that is, within a clinically relevant concentration.

Currently, multiple commercial toxin B ELISA diagnostic kits are available in the market and offer sensitive detection of toxin B at nanograms/mL concentrations. It is to be noted that the current clinical usage of the unmodified ssDNA MRE identified in this study is limited due to its lower sensitivity. However, ssDNA MREs have several advantages over antibodies, such as inexpensive chemical synthesis and reusability [52]. The use of the toxin B-specific MRE in the modified sandwich ELISA assay therefore has a cost advantage over other currently available diagnostic techniques and may offer an option for rapid initial screening of CDI. This MRE may also be incorporated into an SPR biosensor for real-time, label-free toxin B detection in biological matrices [42, 44]. It is also possible to increase the stability of the MRE through chemical modification to bases of DNA and that may stabilize its secondary structure in complex matrices [53]. Overall, the use of R12.69 demonstrated a proof of concept in substituting antibody as the antigen capturing element in clinically relevant samples and may have the potential to augment current and emerging diagnostic techniques of* C. difficile* infections.

## 4. Conclusions

A ssDNA molecular recognition element specific for toxin B has been identified with nanomolar affinity after twelve rounds of selection and is highly specific toward the target of interest. It also showed sensitive detection of toxin B in human fecal sample through a modified sandwich ELISA assay and demonstrated a proof-of-concept diagnostic application of the ssDNA MRE.

## Figures and Tables

**Table 1:** Systematic evolution of ligands by exponential enrichments (SELEX) scheme for toxin B-specific MRE selection.

Round	Positive selection	Time	Negative selection	Time
1	Immobilized target (IT)	24 hrs	—	—
2	IT	18 hrs	BSA INT	22 hrs
3	IT	13 hrs	BSA INT	26 hrs
4	IT	7 hrs	Exotoxin A INT	22 hrs
5	IT	3 hrs	Exotoxin A INT	26 hrs
6	IT	30 min	BSA INT	24 hrs
7	IT/competitive elution with 20 *μ*g/mL free toxin B	5 min/5 min	IT/competitive elution with 20 *μ*g/mL free BSA	5 min/5 min
8	IT/competitive elution with 20 *μ*g/mL free toxin B	5 sec/5 sec	IT/competitive elution with 20 *μ*g/mL free alpha toxin, 1 hour	5 sec/1 hrs
9	IT/competitive elution with 10 *μ*g/mL free toxin B	5 sec/5 sec	IT/competitive elution with 20 *μ*g/mL free cholera toxin, 1 hour	5 sec/1 hrs
10	IT/competitive elution with 5 *μ*g/mL free toxin B	5 sec/5 sec	IT/competitive elution with 20 *μ*g/mL free exotoxin A, 1 hour	5 sec/1 hrs
11	IT/competitive elution with 2.5 *μ*g/mL free toxin B	5 sec/5 sec	IT/competitive elution with 20 *μ*g/mL free BSA, 24 hrs	5 sec/24 hrs
12	IT/competitive elution with 1 *μ*g/mL free toxin B	5 sec/5 sec	—	

*In vitro* selection performed for identifying toxin B-specific MRE. Immobilized target (IT) is toxin B conjugated to magnetic beads. Immobilized negative target (INT) is negative targets conjugated to magnetic beads. BSA is the abbreviation for bovine serum albumin. Times listed are incubation times in hours (hrs), minutes (min), or seconds (sec).

**Table 2:** Cross binding reactivity of R12.69 ssDNA MRE.

Target	Average fluorescence (RFU)	Standard deviation	*P* value	Selectivity ratio
Toxin B	0.0176	0.0066	—	—
Cholera toxin	0.0080	0.0041	0.0497	2.2
Alpha toxin	0.0033	0.0022	0.0117	5.4
Exotoxin A	0.0037	0.0022	0.0130	4.7
Bovine serum albumin	0.0030	0.0018	0.0106	5.8

For each protein target, average fluorescence is given with standard deviation. The *P* value is given from a *t*-test between toxin B and other negative targets. The selective ratio describes the number of times greater binding to toxin B than to other negative targets.
